# First-principles insights into the Janus MoPC monolayer as a promising anode for sodium-ion batteries

**DOI:** 10.1039/d5ra09561a

**Published:** 2026-03-18

**Authors:** Tuan V. Vu, Duc-Quang Hoang, Thi H. Ho, Hien D. Tong, Khang D. Pham

**Affiliations:** a Laboratory for Computational Physics, Institute for Computational Science and Artificial Intelligence, Van Lang University Ho Chi Minh City Vietnam tuan.vu@vlu.edu.vn; b Faculty of Mechanical, Electrical, and Computer Engineering, Van Lang School of Technology, Van Lang University Ho Chi Minh City Vietnam; c Faculty of Applied Sciences, HCMC University of Technology & Engineering 01 Vo Van Ngan, Thu Duc Ho Chi Minh City 700000 Vietnam; d Faculty of Engineering, Vietnamese-German University (VGU) Ring road 4, Quarter 4, Thoi Hoa Ward Ho Chi Minh City Vietnam; e Institute of Research and Development, Duy Tan University Da Nang Vietnam phamdinhkhang@duytan.edu.vn; f School of Engineering & Technology, Duy Tan University Da Nang Vietnam

## Abstract

The development of high-performance anode materials is critical for advancing next-generation sodium-ion batteries. Using comprehensive density–functional–theory calculations, we reveal that the Janus MoPC monolayer possesses a notable combination of structural stability, fast ion transport, and high Na storage capability. Specifically, the proposed MoPC monolayer is predicted to exhibit robust mechanical and thermal stability together with intrinsic metallic conductivity. Na adsorption is energetically favorable across a wide range of coverages, and Na^+^ migration proceeds with an ultralow barrier of 0.06 eV along the most preferred pathway, suggesting favorable intrinsic diffusion under idealized conditions. The monolayer can accommodate up to six Na layers with a low and relatively stable calculated open-circuit voltage profile, yielding a high theoretical capacity of 1157.46 mAh g^−1^. Crucially, the MoPC monolayer preserves its metallic character even at high sodiation levels, ensuring electronic conductivity throughout battery operation. Overall, our findings indicate that Janus MoPC is a viable two-dimensional anode candidate for sodium-ion batteries with high Na storage capability and intrinsically favorable Na transport.

## Abstract 2

MoPC monolayer as a promising anode for SIBs with a theoretical capacity of 1157.46 mAh g^−1^ and low diffusion barrier of 0.06 eV.

## Graphical abstract

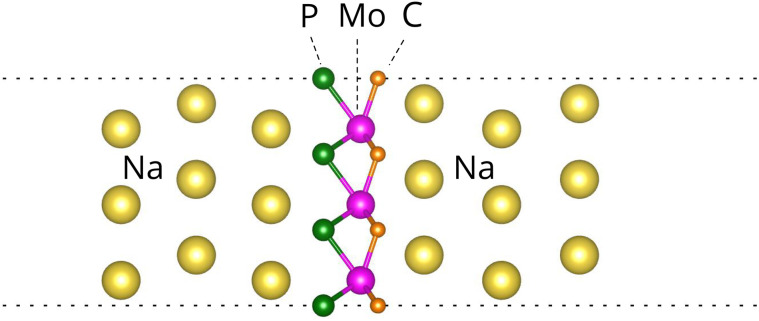

## 1 Introduction

The rapid expansion of large-scale energy storage systems and portable electronics has intensified the development of rechargeable batteries that not only demand high performance but also remain economically and environmentally sustainable. Lithium-ion batteries (LIBs) currently dominate the market owing to their high energy density and technological maturity; however, increasing concerns regarding lithium scarcity, uneven resource distribution, rising production costs, and safety risks have highlighted their long-term limitations.^1–4^ These issues underscore the urgent need to explore alternative energy-storage technologies based on more abundant and cost-effective materials.

Sodium-ion batteries (SIBs) have recently gained attention as a promising next-generation energy-storage technology because sodium is inexpensive, widely distributed, and shares similar intercalation chemistry with lithium, making many LIB design principles transferable to SIBs.^5,6^ Although the larger ionic size and greater mass of Na^+^ introduce challenges related to diffusion kinetics and structural distortion, SIBs remain highly attractive for grid-level and stationary energy storage applications where material cost and scalability outweigh size and weight concerns.^7^

In sodium-ion batteries, as in other rechargeable systems, the electrochemical performance is strongly dictated by the characteristics of the electrode materials. In particular, the anode strongly influences the achievable specific capacity, rate capability, coulombic efficiency, and long-term cycling stability.^8,9^ An effective SIB anode must provide robust structural integrity under repeated Na insertion/extraction, exhibit moderate Na adsorption energies, maintain low diffusion barriers, and deliver adequate electrical conductivity and operating voltage.^10^ Numerous anode materials have been explored, yet many face persistent challenges such as sluggish Na-ion diffusion, volume expansion, and structural degradation.^8,9,11^

In recent years, two-dimensional (2D) materials have attracted broad interest owing to their atomic-scale thickness, high density of accessible sites, and adjustable chemical properties, which collectively promote Na adsorption, diffusion, and reversible storage.^12–16^ Among emerging 2D candidates, the Janus MoPC monolayer has recently drawn attention due to its structurally and electronically distinctive characteristics. MoPC crystallizes in a hexagonal lattice with space group *P*3*m*1, where Mo atoms are asymmetrically sandwiched between chemically different P and C layers, forming inequivalent Mo_3_P_3_ and Mo_3_C_3_ honeycomb motifs and generating a pronounced out-of-plane dipole moment.^17,18^ First-principles studies have demonstrated that MoPC possesses exceptional intrinsic stability, including a large cohesive energy of 6.27 eV per atom, dynamically stable phonon spectra, and robust mechanical and thermal behavior.^17^ The material also exhibits metallic conductivity dominated by Mo-d and P/C-p orbitals, while its polar surface and mixed ionic–covalent bonding environment produce a high density of chemically active sites, enabling excellent hydrogen evolution reaction activity.^17^ With those excellent metallic characteristics based on the robust structural behavior with asymmetric charge distribution and chemically active surfaces, such properties are highly desirable for Na-ion storage. This hints that Janus MoPC structures may serve as a compelling anode material for SIBs. However, despite its promising profile, the Na adsorption behavior, diffusion kinetics, and overall electrochemical properties of MoPC remain completely unexplored.

In this work, we investigate several key properties of the Janus MoPC monolayer using first-principles density functional theory (DFT), with the aim of evaluating its potential as an anode material for sodium-ion batteries. Our study examines its structural stability under various sodiation configurations, Na adsorption energies and preferred binding sites, electronic structure evolution upon sodiation, charge redistribution characteristics, Na-ion migration pathways using climbing-image nudged elastic band (CI-NEB) calculations, average open-circuit voltage (OCV), and theoretical specific capacity. These results deepen the fundamental understanding of Na-storage mechanisms on Janus MoPC and provide critical insights into its feasibility as a high-performance anode for next-generation sodium-ion batteries.

## 2 Computational details

All calculations were performed using the QUANTUM ESPRESSO package^19^ based on density functional theory. The electron-ion interactions were described using projector augmented-wave (PAW) pseudopotentials,^20^ while the exchange–correlation energy was treated with the generalized gradient approximation in the PBE form (GGA–PBE).^21^ In addition, van der Waals interactions were included using the DFT-D3 dispersion correction. A plane-wave kinetic-energy cutoff of 520 eV was adopted. A vacuum spacing of at least 15 Å was introduced perpendicular to the MoPC layer to avoid spurious periodic interactions, and the Brillouin zone was sampled using a *Γ*-centered Monkhorst–Pack mesh.^22^ For structural relaxations of the 3 × 3 supercells, a 4 × 4 × 1 *k*-point mesh was used, whereas a denser 6 × 6 × 1 mesh was employed for total-energy self-consistent calculations and for the subsequent electronic–structure analyses, including band structures and the density of states. Structural optimization was carried out until the residual forces on atoms were less than 0.01 eV Å^−1^ and the electronic self-consistency threshold reached 10^−6^ eV. For Na adsorption calculations, Na atoms were placed on the MoPC surfaces and the entire Na/MoPC system was fully relaxed within DFT through total-energy minimization.

## 3 Results and discussion

**Fig. 1:**
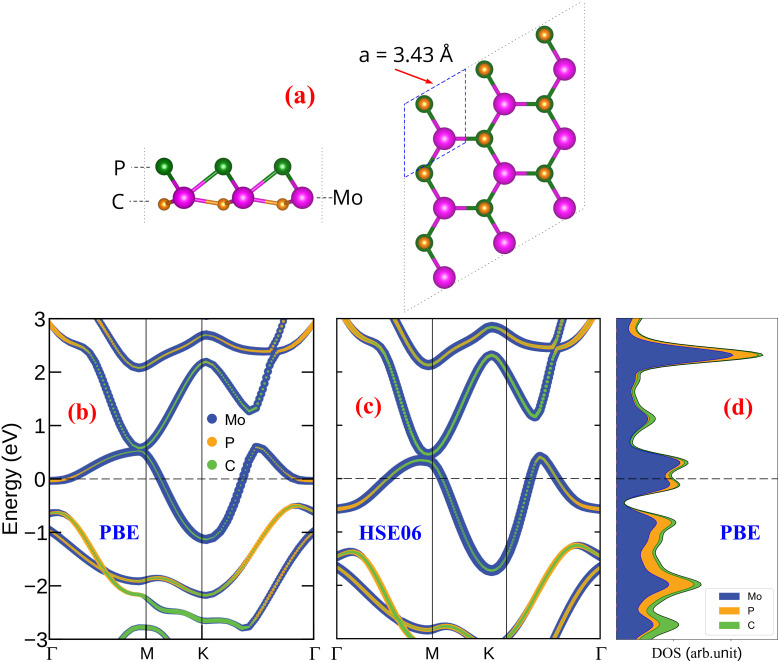
(a) Side and top views of the relaxed MoPC monolayer, (b, and c) atom-projected band structures computed with PBE and HSE06 (HSE06 single-point on the PBE geometry), and (d) atom-projected PDOS computed with PBE.

The fully optimized structure of the Janus MoPC monolayer is shown in Fig. 1(a). Our calculations indicate that MoPC adopts a trigonal lattice with an in-plane lattice constant of *a* = 3.43 Å, which is consistent with the previously reported value of 3.45 Å.^18^ The computed Mo–P and Mo–C bond lengths are 2.53 Å and 2.01 Å, respectively, in close agreement with the reported values of 2.53 Å and 2.02 Å.^17^ These consistencies support the reliability of the optimized structural model used in this study.

The electronic band structure and density of states (DOS), shown in Fig. 1(b) and (d), reveal that the Janus MoPC monolayer exhibits intrinsic metallic behavior. Multiple bands cross the Fermi level, and a finite DOS at *E*_F_ is clearly observed. To further validate the metallic character beyond GGA-PBE, we also calculated the projected band structure using the HSE06 functional, as shown in Fig. 1(c) (single-point on the PBE-relaxed geometry). The HSE06 result likewise displays dispersive bands crossing *E*_F_ without opening a band gap, indicating that the metallicity is preserved. Compared with the PBE band structure in Fig. 1(b), HSE06 mainly induces a slight shift of band energies while maintaining the overall band topology around the Fermi level. This intrinsic metallicity implies high electronic conductivity, a crucial requirement for fast charge transport when MoPC is employed as an anode material for sodium-ion batteries.

**Fig. 2:**
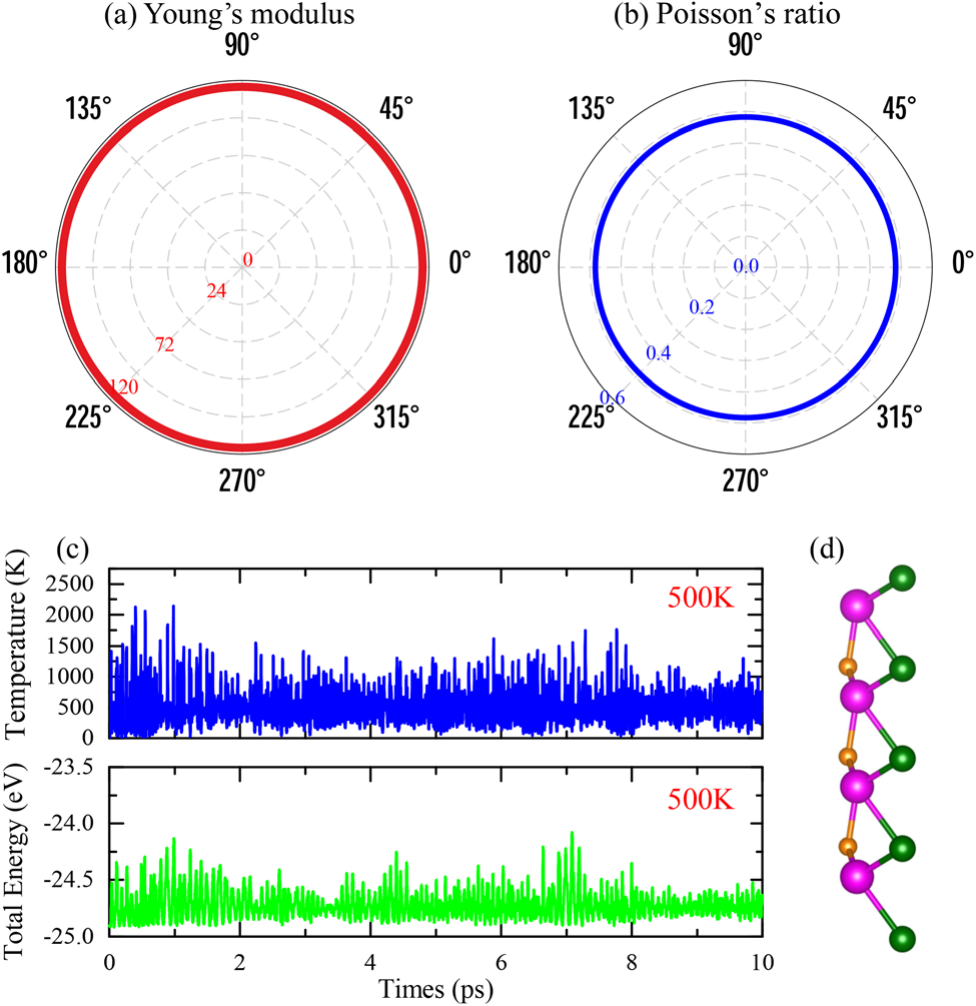
(a, and b) The angular dependences of the in-plane Young's modulus (unit in N m^−1^) and Poisson's ratio (scalar quantity), (c) *ab initio* molecular dynamics (AIMD) results at 500 K showing the temporal evolution of temperature and total energy, and (d) the crystal structures of the Janus MoPC monolayer at the end of the AIMD test.

To assess the mechanical stability of the MoPC monolayer, we calculated its in-plane elastic constants. The obtained values, *C*_11_ = 151.09 N m^−1^, *C*_12_ = 72.90 N m^−1^, and *C*_66_ = 39.09 N m^−1^, satisfy the Born stability criteria for two-dimensional hexagonal crystals (*C*_11_ > 0 and *C*_11_ > |*C*_12_|^23^). These results suggest that the MoPC monolayer is mechanically stable under small in-plane perturbations. In addition, Fig. 2(a) and (b) show the angular dependence of the in-plane Young's modulus and Poisson's ratio. The circular contour of the Young's modulus, with an value of 115.91 N m^−1^, indicates that the MoPC monolayer exhibits isotropic elastic behavior. This arises from the Mo–P–C bonding arrangement within the structure. The Poisson's ratio, with a representative value of 0.48, further supports that the material can withstand moderate deformation without signs of mechanical instability.

The thermal stability of MoPC was examined using *ab initio* molecular dynamics (AIMD) simulations at 500 K for a total simulation time of 10 ps. As shown in Fig. 2(c), the system temperature fluctuates around the intended value, and the total energy exhibits only bounded fluctuations without an apparent drift, indicating the absence of thermal runaway or structural instability within the simulated time window. Representative snapshots in Fig. 2(d) show that the lattice framework is well retained during the AIMD run, and no bond breaking, reconstruction, or noticeable lattice distortion is observed. These observations suggest that the pristine Janus MoPC monolayer remains thermally stable at 500 K on the AIMD timescale, consistent with the thermal-stability assessment reported in ref. 17.

**Fig. 3:**
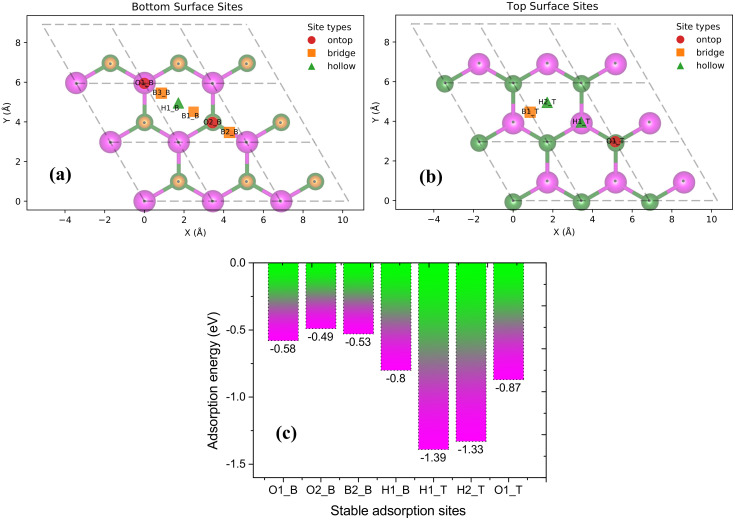
(a) Bottom-surface adsorption sites (O1_B, O2_B, B1_B, B2_B, B3_B, and H1_B) and (b) top-surface adsorption sites (H1_T, H2_T, B1_T, and O1_T) of the Janus MoPC monolayer, illustrating ten high-symmetry Na adsorption positions. (c) Adsorption energies of the energetically favorable configurations after full structural relaxation. Here, the subscripts B and T denote the bottom (C-terminated) and top (P-terminated) surfaces, respectively, while O, B, and H refer to on-top, bridge, and hollow sites.

To systematically evaluate the Na storage behavior of Janus MoPC, we performed a layer-by-layer Na adsorption analysis on a 3 × 3 MoPC supercell. First, a single Na atom was placed at all high-symmetry adsorption sites on both the bottom (C-terminated) and top (P-terminated) surfaces of the MoPC monolayer, as illustrated in Fig. 3(a) and (b), respectively, generating a total of ten initial configurations. All configurations were fully relaxed, and the most stable adsorption site was identified as the one with the most negative adsorption energy. Based on this optimal position, the first Na layer was constructed by adding eight additional Na atoms at symmetry-equivalent sites within the 3 × 3 supercell, followed by full structural optimization. The same procedure was then applied to determine the second Na layer: a Na atom was sequentially placed at all possible adsorption sites on both surfaces of the fully sodiated first-layer structure. The most stable configuration was selected, and eight additional Na atoms were added at symmetry–equivalent positions to complete the second layer containing nine Na atoms in total. This iterative adsorption procedure was continued until no additional Na adsorption sites with negative adsorption energy could be identified on either surface, indicating that further Na insertion becomes thermodynamically unfavorable. The resulting structure was thus defined as the saturated configuration, corresponding to the maximum Na adsorption capacity achievable within the 3 × 3 MoPC supercell under the adopted thermodynamic criterion. In our calculations, saturation is reached at *x* = 6 (Na_6_MoPC), and the sequential Na_*x*_MoPC structures (*x* = 1–6), including the fully saturated configuration on both sides, are explicitly shown in Fig. 7.

To examine the dynamical stability of the Na_6_MoPC configuration, AIMD simulations were carried out for 5 ps. As shown in Fig. S1 (SI), the temperature fluctuates around 300 K and the total energy exhibits only small oscillations without any noticeable drift, indicating stable equilibration. Moreover, the Mo–P–C framework remains intact throughout the simulation, with no bond breaking or severe structural reconstruction observed. The atomic fluctuations are mainly associated with the outermost adsorbed Na atoms on both sides due to their weak ionic interactions and thermal motion. These results confirm that Na_6_MoPC is dynamically stable at room temperature.

The average adsorption energy for adding a new set of Na atoms was calculated using:
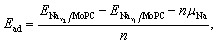
where 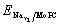 and 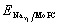 are the total energies of the system after and before adding the Na atoms, respectively. Here, *n* denotes the number of Na atoms introduced in the new layer, and *µ*_Na_ is the chemical potential of metallic Na. A negative value of *E*_ad_ indicates that the addition of the new Na layer is thermodynamically favorable, whereas *E*_ad_ ≥ 0 implies that further Na adsorption is no longer energetically preferable.

The structural optimizations of the ten initial Na adsorption configurations on the MoPC monolayer reveal that seven of them are energetically stable, with their adsorption energies shown in Fig. 3(c). Among these, the H1_T site exhibits the most negative adsorption energy (−1.39 eV), identifying it as the most favorable adsorption position for Na atoms. The relatively large negative adsorption energy indicates that Na binds stably to the MoPC surface, which is an important characteristic for potential anode materials in sodium-ion batteries.

**Fig. 4:**
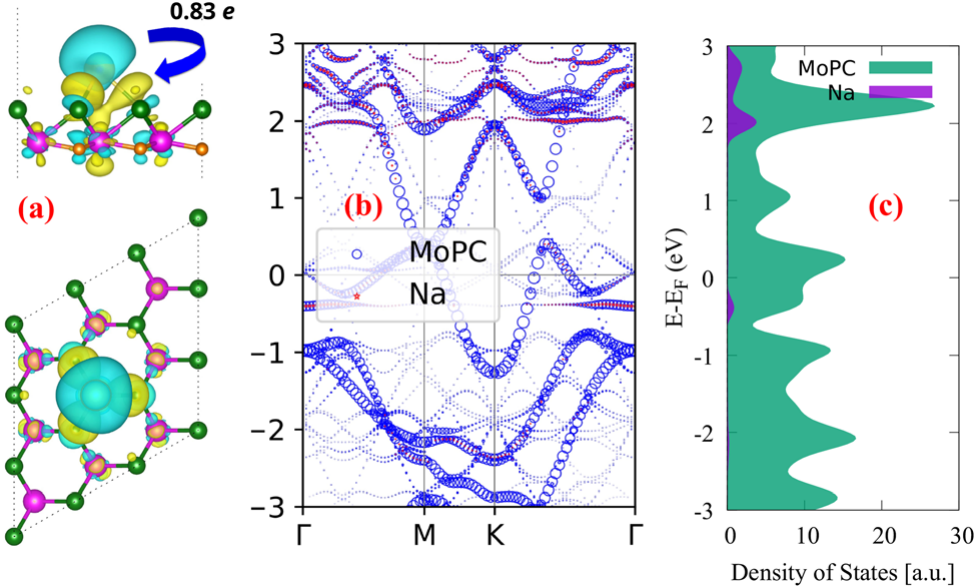
(a) Charge density difference of the most stable single-Na-adsorbed configuration on a 3 × 3 MoPC supercell. The yellow and cyan isosurfaces represent charge accumulation and charge depletion, respectively, with an isosurface value of 0.001 e Å^−3^, (b) unfolding electronic band structure, and (c) atom-projected partial density of states of the same configuration.

Based on the most stable adsorption geometry at H1_T adsorption site, we performed the charge density difference (CDD), unfolding band structure, and projected density of states (PDOS) analyses, as shown in Fig. 4. The CDD was computed using the expression:

1 Δ*ρ* = *ρ*_MoPC+Na_ − *ρ*_MoPC_ − *ρ*_Na_,

where *ρ*_MoPC+Na_ is the charge density of the Na-adsorbed system, and *ρ*_MoPC_ and *ρ*_Na_ are the charge densities of the isolated monolayer and isolated Na atom, respectively.

The charge density difference (CDD) images shown in Fig. 4(a) reveal a redistribution of electronic charge upon Na adsorption, where electrons are transferred primarily from the Na atom to the MoPC monolayer. The calculated charge transfer of approximately 0.83e indicates that Na is largely ionized and acts as an electron donor. This suggests that the MoPC surface has a good ability to accommodate the donated charge, which is beneficial for electrochemical stability during Na adsorption. The weighted band structure shown in Fig. 4(b) further illustrates the electronic response to adsorption. Although some band renormalization appears near the Fermi level due to the presence of Na, several dispersive bands continue to cross the Fermi level, indicating that the metallic character of MoPC is preserved. This implies that Na adsorption does not substantially diminish the intrinsic electrical conductivity of the monolayer, an important requirement for anode materials. To further confirm this conclusion beyond GGA-PBE, the HSE06 band structure of the most stable Na-adsorbed configuration is provided in Fig. S2 (SI), which also shows bands crossing the Fermi level without opening a band gap. The PDOS results in Fig. 4(c) support this behavior. A finite density of states remains at the Fermi level after Na adsorption, and new hybridized features emerge due to interactions between the Na 3s and Mo 4d orbitals. These features reflect the electronic coupling between Na and MoPC and show that the monolayer retains conductive properties in the adsorbed state.

**Fig. 5:**
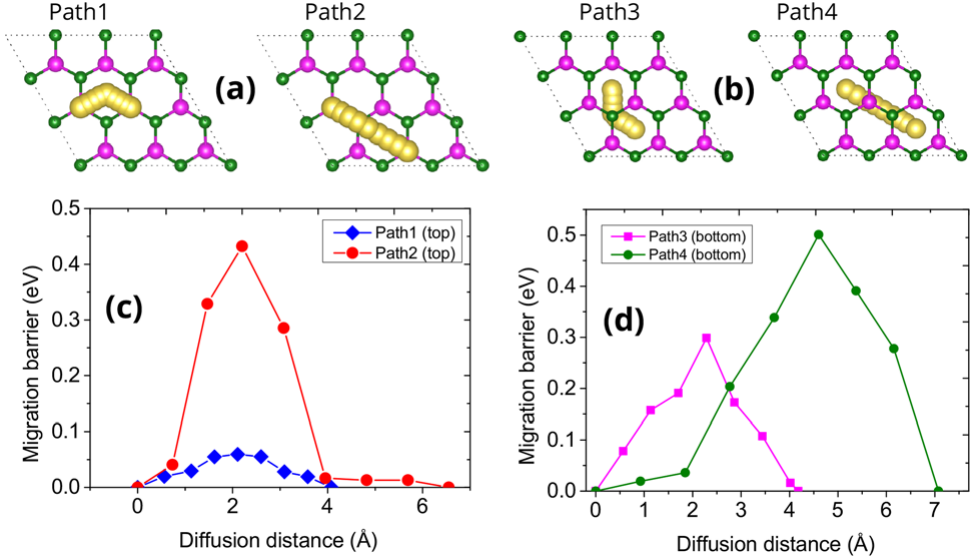
(a, and b) Possible Na migration pathways on the top (Path 1, Path 2) and bottom surfaces (Path 3, Path 4) of the Janus MoPC monolayer. (c, and d) Corresponding Na diffusion energy profiles obtained from CI–NEB calculations along the four pathways.

To evaluate the ionic mobility of Na on the Janus MoPC monolayer, we investigated four representative diffusion pathways, as illustrated in Fig. 5(a) and (b). Therein, Paths 1 & 2 correspond to migration along the top surface, and Paths 3 & 4 describe Na diffusion across the bottom surface. These pathways were selected based on the connectivity between the most energetically favorable adsorption sites identified earlier.

The calculated CI-NEB diffusion energy profiles for the four pathways are presented in Fig. 5(c) and (d). On the top surface, Na migration along Path 1 shows a relatively low energy barrier of 0.06 eV, indicating that ion movement between adjacent hollow sites is energetically favorable. In comparison, Path 2 exhibits a higher barrier of around 0.43 eV, likely due to the presence of less favorable intermediate configurations. These differences suggest that Na diffusion on the Mo-terminated surface may display directional dependence, with Path 1 offering a more favorable migration route under the conditions considered.

On the bottom surface, Na diffusion follows different energetics. Path 3 presents a moderate barrier of 0.30 eV, whereas Path 4 reaches up to 0.50 eV, the highest among all four pathways. The larger migration barriers on the bottom P/C-terminated surface can be attributed to stronger local electrostatic interactions and less favorable geometric coordination for intermediate adsorption states.

Overall, the lowest diffusion barrier obtained for Na migration on the MoPC monolayer is 0.06 eV, indicating that ion transport on this surface is energetically favorable. Such a low barrier suggests that Na ions can migrate efficiently across the monolayer, which is beneficial for applications requiring fast charge–discharge processes in sodium-ion batteries.

Next, we analyze the corresponding open-circuit voltage (OCV) behavior and the evolution of adsorption energetics as the Na concentration increases. The OCV was calculated using the standard thermodynamic expression:^24,25^2
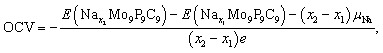
where 
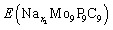
 and 
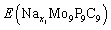
 are the total energies of the system at two adjacent sodiation states with Na contents *x*_2_ and *x*_1_ (*x*_2_ > *x*_1_), respectively. Here, (*x*_2_ − *x*_1_) represents the number of inserted Na atoms between the two states, *µ*_Na_ is the chemical potential of Na approximated by the total energy per atom of bulk bcc Na metal, and *e* is the elementary charge.

**Fig. 6:**
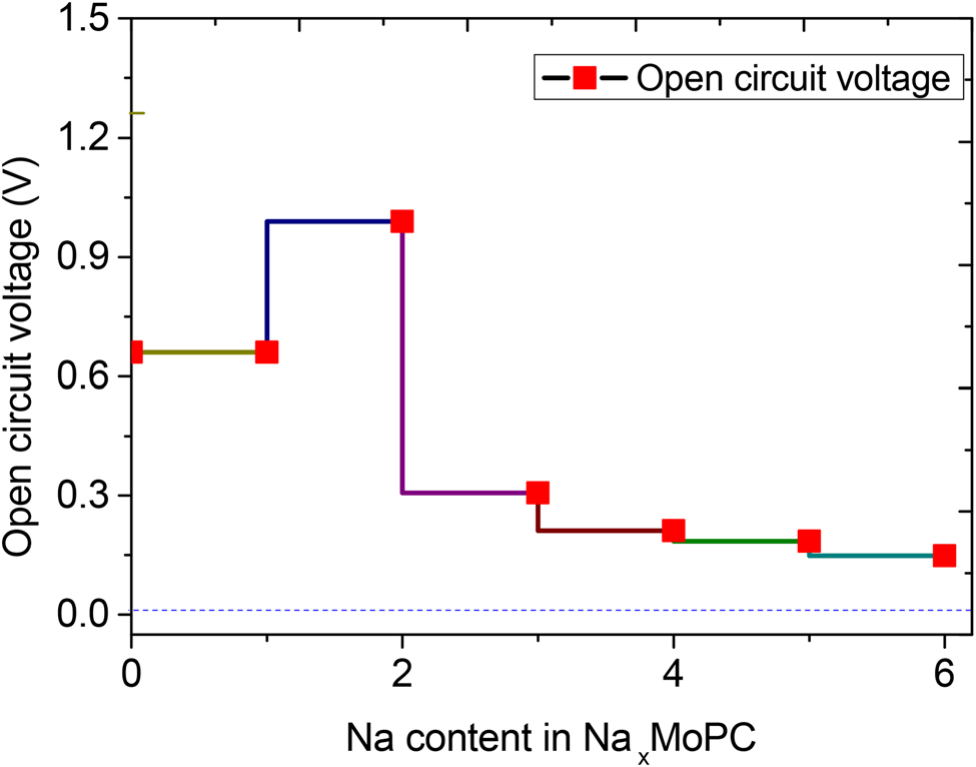
Open-circuit voltage profile of the Na_*x*_MoPC system as a function of Na concentration *x*.

**Fig. 7:**
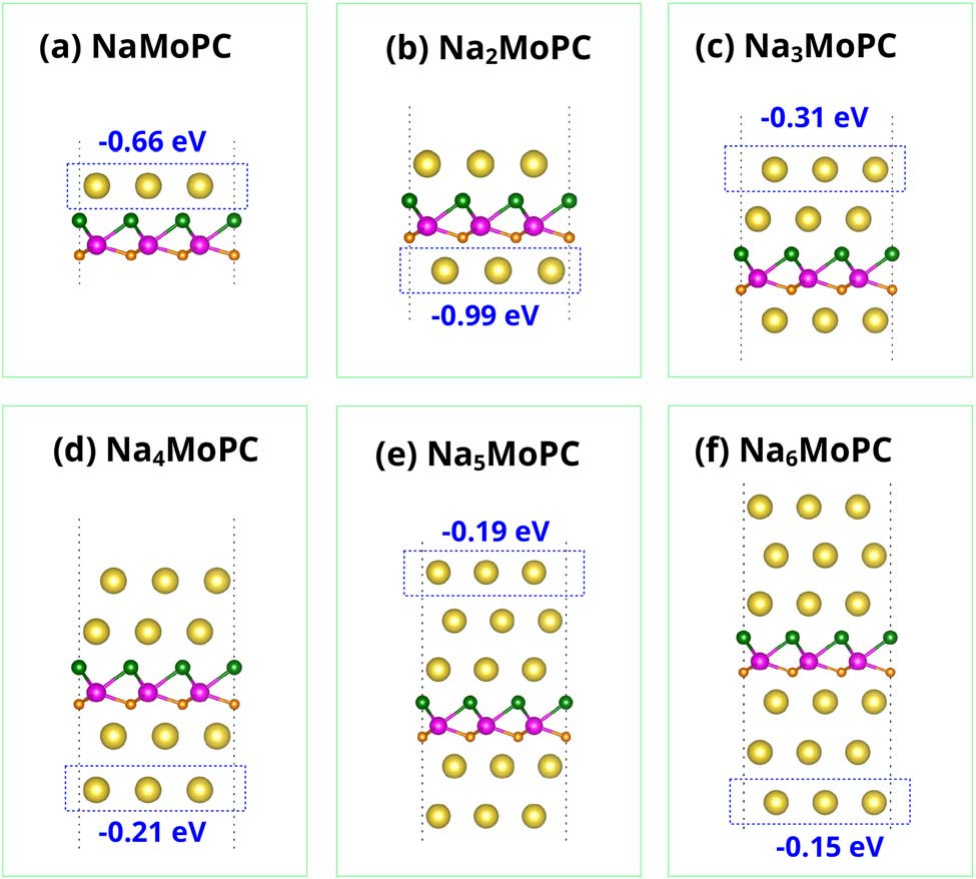
Top and side views of the Na_*x*_MoPC structures: (a) Na_1_MoPC, (b) Na_2_MoPC, (c) Na_3_MoPC, (d) Na_4_MoPC, (e) Na_5_MoPC, and (f) Na_6_MoPC. The average adsorption energies per Na atom (in eV) are indicated beneath each structure.

Fig. 6 presents the OCV profile for Na_*x*_MoPC (3 × 3 supercell, Mo_9_P_9_C_9_), providing insight into the thermodynamic driving force for Na insertion at different stages of sodiation. The fully relaxed adsorption configurations for Na contents from *x* = 1 to 6, along with their average adsorption energies, are shown in Fig. 7, which provides complementary thermodynamic information on the stability of Na incorporation. At the initial stage (*x* = 1), Na atoms preferentially occupy the most stable hollow sites on the P-terminated surface. The OCV at this stage is about 0.66 V (Fig. 6), indicating a strong thermodynamic driving force for the first Na insertion. As the Na content increases to *x* = 2, the OCV reaches a local maximum, consistent with the continued filling of the most favorable adsorption sites. Beyond *x* = 2, the OCV gradually decreases as Na–Na repulsion becomes more significant and the remaining adsorption sites become progressively less favorable. Specifically, the OCV drops to ∼0.31 V at *x* = 3, and further decreases to ∼0.21, 0.19, and 0.15 V for *x* = 4, 5, and 6, respectively (Fig. 6).

Fig. 7 shows that Na adsorption remains thermodynamically favorable (negative average adsorption energies) throughout *x* = 1–6, while the binding strength becomes progressively weaker at higher Na contents, indicating the onset of a weakly bound Na overlayer at *x* = 6. This trend is consistent with an increased Na-surface distance and a more delocalized charge distribution at high coverages.

Based on the maximum Na loading considered here (*x* = 6), *i.e.*, the Na_6_MoPC configuration (Fig. 7(f)), we estimated the theoretical Na storage capacity of Janus MoPC. The specific capacity *C* was calculated using:^26^3
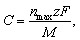
where *n*_max_ = 6 is the maximum number of Na atoms per MoPC formula unit, *z* = 1 is the valence charge of each Na ion, *F* = 26801 mAh mol^−1^ is the Faraday constant, and *M* = 138.93 g mol^−1^ is the molar mass of pristine MoPC. Substituting these values yields a theoretical capacity of 1157.46 mAh g^−1^. This value is notably higher than many established two-dimensional anode materials for sodium-ion batteries. For example, MXene Ti_3_C_2_T_*x*_, Mo_2_CO_2_, Janus MoSSe, and C_6_BN exhibit capacities of approximately 125,^27^ 379,^28^ 510,^29^ and 553 mAh g^−1^,^30^ respectively. Other recently proposed high-capacity 2D monolayers-such as SiC_2_ (1203 mAh g^−1^),^31^ BSi (1034 mAh g^−1^),^32^ and C_6_B_4_ (1395 mAh g^−1^)^33^-offer comparable or higher capacities but typically exhibit larger Na diffusion barriers, with reported values of 0.28 eV, 0.24 eV, and 0.123 eV, respectively. The favorable capacity of MoPC is attributed to its ability to host multiple Na layers while maintaining stable adsorption energetics.

**Fig. 8:**
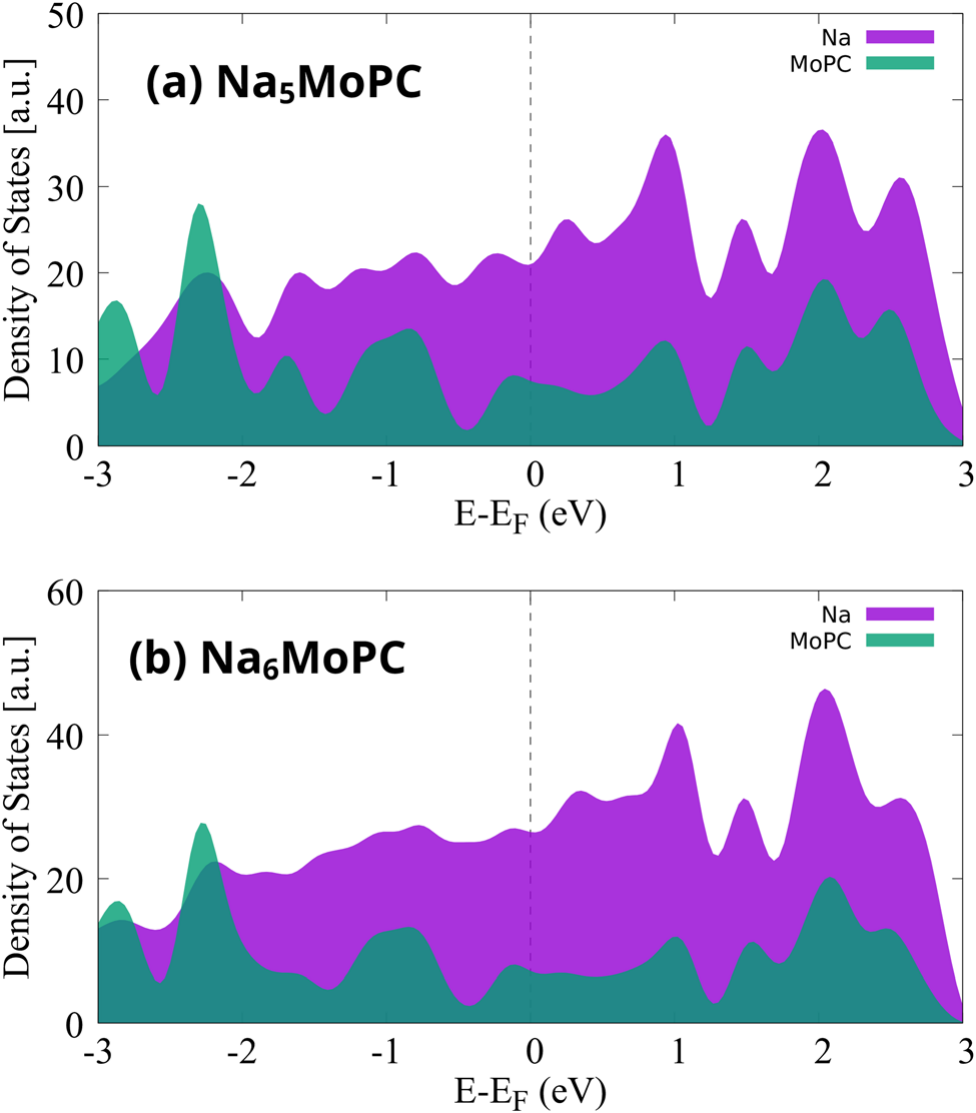
PDOS of highly sodiated MoPC at (a) Na_5_MoPC and (b) Na_6_MoPC coverage, showing the contributions from Na atoms (purple) and the MoPC monolayer (green).

To examine whether MoPC preserves its metallic nature at high Na concentrations, we analyzed the projected density of states (PDOS) for the highly sodiated Na_5_MoPC and Na_6_MoPC structures (Fig. 8). In both cases, a finite density of electronic states persists at the Fermi level, indicating that the monolayer remains metallic even at maximum Na loading. The states near the Fermi level are primarily derived from Na orbitals, supplemented by contributions from the MoPC framework. This suggests that adsorbed Na layers introduce additional carriers that sustain, and may enhance, the electronic conductivity of the system.

**Fig. 9:**
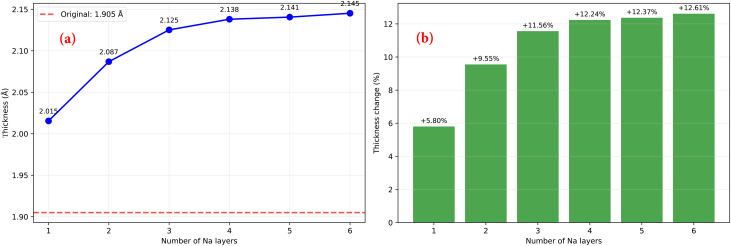
(a) Thickness evolution of the MoPC monolayer upon Na adsorption as a function of Na coverage (*x* = 1–6) and (b) the corresponding thickness expansion ratio relative to pristine MoPC monolayer.

To evaluate the structural stability of MoPC during Na storage, the thickness variation of the monolayer was analyzed for Na coverages from *x* = 1 to 6, as shown in Fig. 9. The pristine MoPC monolayer has an initial thickness of 1.905 Å. Upon Na adsorption, the thickness gradually increases with increasing Na concentration, reaching 2.015, 2.087, 2.125, 2.138, 2.141, and 2.145 Å for *x* = 1–6, respectively. This corresponds to thickness expansions of approximately 5.80%, 9.55%, 11.56%, 12.24%, 12.37%, and 12.61%. Notably, the expansion increases progressively at low Na concentrations and then tends to saturate at higher coverages, indicating that structural deformation becomes moderate once the most favorable adsorption sites are occupied. The maximum thickness expansion of approximately 12.6% at full saturation suggests that the MoPC framework maintains good structural integrity during Na insertion.

## 4 Conclusion

In summary, our first-principles calculations predict that the Janus MoPC monolayer exhibits a combination of properties desirable for sodium-ion battery anodes. The material shows stable mechanical behavior and AIMD-supported thermal robustness, and possesses intrinsic metallic conductivity. Na-ion migration on MoPC proceeds with an ultralow intrinsic diffusion barrier of 0.06 eV along the most favorable pathway, suggesting favorable surface diffusion under the idealized conditions of our calculations. The computed adsorption energetics indicate that MoPC can accommodate multiple Na layers on both sides of the monolayer while retaining favorable adsorption strengths and a low and relatively stable calculated open-circuit voltage profile across the sodiation range. At full Na loading, the Na_6_MoPC configuration yields a theoretical capacity of 1157.46 mAh g^−1^, which is comparable to or higher than that of several reported two-dimensional anode materials. Electronic structure calculations confirm that MoPC remains metallic upon heavy sodiation, implying sustained electronic conductivity over the considered Na loading range. Overall, these results suggest that Janus MoPC is a promising candidate for further exploration as a sodium-ion battery anode, and they provide a theoretical basis for potential experimental validation and material development.

## Conflicts of interest

There are no conflicts of interest to declare.

## Supplementary Material

RA-016-D5RA09561A-s001

## Data Availability

The data supporting this article are provided in the supplementary information (SI). Supplementary information: AIMD simulation results, HSE06 band structure calculations, and the complete optimized geometric coordinates of the calculated structures. See DOI: https://doi.org/10.1039/d5ra09561a.
