# Supplementary material for: First-principles insights into the Janus MoPC monolayer as a promising anode for sodium-ion batteries
Source: RSC Adv. 2026 Mar 18;16(16):14951–8. doi: 10.1039/d5ra09561a (PMC12997422; doi:10.1039/d5ra09561a)
Supplement: RA-016-D5RA09561A-s001 [file RA-016-D5RA09561A-s001.pdf]

Supplementary Information (SI) for RSC Advances.
This journal is © The Royal Society of Chemistry 2026

Supplementary Information for "First-principles insights into the Janus MoPC monolayer as a promising anode for sodium-ion batteries"

Tuan V. Vu[1,2], Duc-Quang Hoang[3], Thi H. Ho[1,2], Hien D. Tong[4], Khang D. Pham[5,6,*]
[1]*Laboratory for Computational Physics, Institute for Computational Science and Artificial Intelligence, Van Lang University, Ho Chi Minh City, Vietnam*
[2]*Faculty of Mechanical - Electrical and Computer Engineering, School of Technology, Van Lang University, Ho Chi Minh City, Vietnam*
[3]*Faculty of Applied Sciences, HCMC University of Technology & Engineering, 01 Vo Van Ngan, Thu Duc, Ho Chi Minh City 700000, Vietnam*
[4]*Faculty of Engineering, Vietnamese-German University (VGU), Ring road 4, Quarter 4, Thoi Hoa Ward, Ho Chi Minh City, Vietnam*
[5]*Institute of Research and Development, Duy Tan University, Da Nang, Vietnam. Email: phamdinhkhang@dtu.edu.vn*
[6]*School of Engineering & Technology, Duy Tan University, Da Nang, Vietnam*

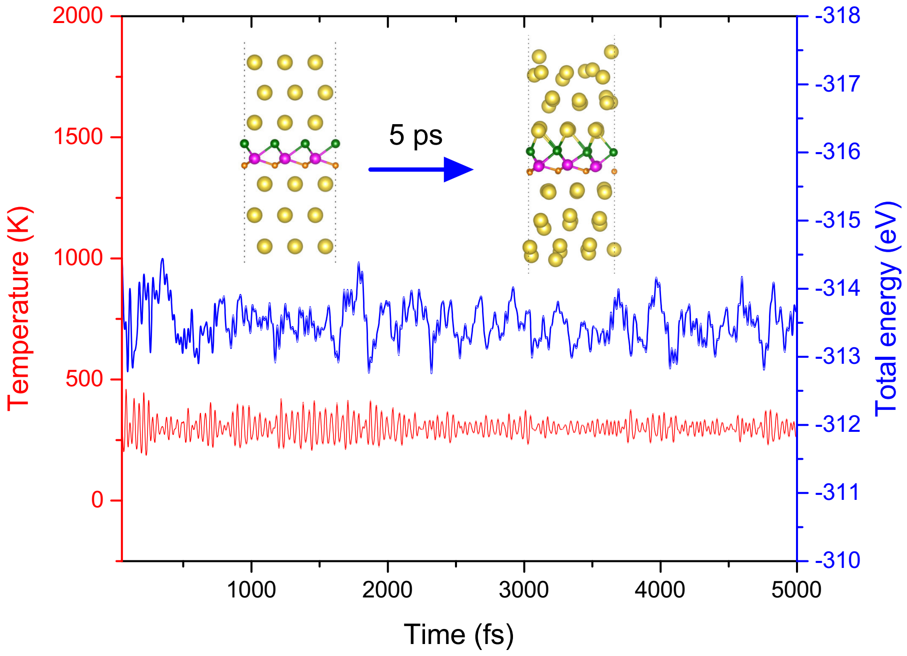

FIG. S1: Ab initio molecular dynamics (AIMD) simulation results for the Na_6MoPC configuration at 300 K over 5 ps. The temperature fluctuates around 300 K and the total energy exhibits only small oscillations without noticeable drift, indicating dynamical stability within the simulated time window.

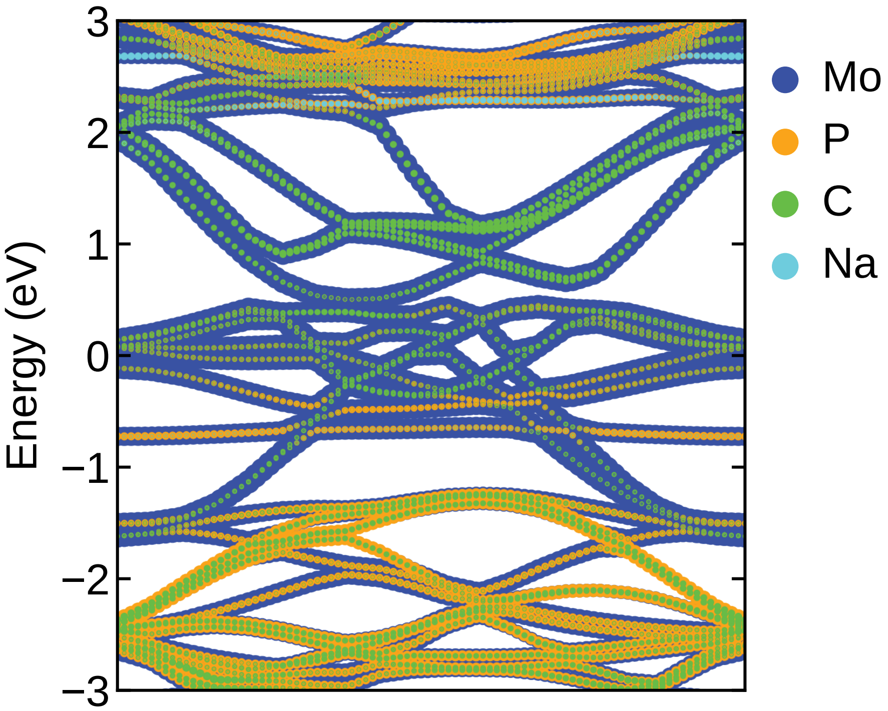

FIG. S2: Atom-projected electronic band structure of the most stable single-Na-adsorbed configuration on a 3×3 MoPC supercell calculated using the HSE06 hybrid functional (single-point on the PBE-relaxed geometry). The Fermi level is set to 0 eV, and bands are observed to cross the Fermi level without opening a band gap.

Optimized structure of MoPC Monolayer

```
C Mo P
   1.00000000000000
     3.4309043455809851   -0.0000012739726676    0.0000004394529628
    -1.7154532760831864    2.9712496842412888   -0.0000004394529628
     0.0000020854992214   -0.0000012040635369   16.5679325707707576
   C    Mo   P
     1     1     1
Selective dynamics
Direct
  0.0000003600948803 -0.0000003600948803  0.4551960813479345
  0.6666665090693868  0.3333334909306132  0.4750241511733270
  0.0000001308357297 -0.0000001308357297  0.5697797674787385

  0.00000000E+00  0.00000000E+00  0.00000000E+00
  0.00000000E+00  0.00000000E+00  0.00000000E+00
  0.00000000E+00  0.00000000E+00  0.00000000E+00
```

Optimized structure of the 3x3 MoPC supercell

```
Mo9 P9 C9
   1.00000000000000
    10.2927129999999991   -0.0000040000000000    0.0000010000000000
    -5.1463599999999996    8.9137489999999993   -0.0000010000000000
     0.0000020000000000   -0.0000010000000000   16.8984169999999985
   C    Mo   P
     9     9     9
Selective dynamics
Direct
  0.0000002139200886 -0.0000002277934878  0.4562237287741340
 -0.0000187418267994  0.3334229735287437  0.4559428830618810
 -0.0000190936092889  0.6665580209044750  0.4559428138900239
  0.3334419709746959  0.0000190860364611  0.4559428327840584
  0.3334421008764109  0.3334231663438326  0.4559429343068491
  0.3333330206927324  0.6666669748321219  0.4560347492802957
  0.6665770355743239  0.0000187422292593  0.4559428931364568
  0.6666668196157369  0.3333331961992114  0.4560437578464378
  0.6665768419822943  0.6665578983856778  0.4559429317571581
  0.2222235792197724  0.1111117738449657  0.4755356419678733
  0.2222086372117372  0.4444168251886560  0.4755162293633751
  0.2222082502580931  0.7777917855881068  0.4755162159964310
  0.5555531586063389  0.1111059846510015  0.4755843461885484
  0.5555532915497555  0.4444467215181910  0.4755843977309577
  0.5555831868954858  0.7777913787575412  0.4755162285946224
  0.8888884752163060  0.1111115065325989  0.4755355654167126
  0.8888940007748938  0.4444468342339066  0.4755843419526416
  0.8888882139699513  0.7777764261787325  0.4755356362894671
 -0.0000000094549946  0.0000000008865321  0.5686737564338262
  0.0000150155503823  0.3330952565570704  0.5684186224747161
  0.0000148474891169  0.6669196574281639  0.5684186180388637
  0.3330803307647466 -0.0000148552749426  0.5684186129109601
  0.3330804178673290  0.3330953254457631  0.5684186580352693
  0.3333332338761955  0.6666667562234114  0.5684603660873445
  0.6669047493461312 -0.0000150189891926  0.5684186217465922
```

```
  0.6666667753373123  0.3333332314578118  0.5684859580174835
  0.6669046773212639  0.6669195791053756  0.5684186579170206

  0.00000000E+00  0.00000000E+00  0.00000000E+00
  0.00000000E+00  0.00000000E+00  0.00000000E+00
  0.00000000E+00  0.00000000E+00  0.00000000E+00
  0.00000000E+00  0.00000000E+00  0.00000000E+00
  0.00000000E+00  0.00000000E+00  0.00000000E+00
  0.00000000E+00  0.00000000E+00  0.00000000E+00
  0.00000000E+00  0.00000000E+00  0.00000000E+00
  0.00000000E+00  0.00000000E+00  0.00000000E+00
  0.00000000E+00  0.00000000E+00  0.00000000E+00
  0.00000000E+00  0.00000000E+00  0.00000000E+00
  0.00000000E+00  0.00000000E+00  0.00000000E+00
  0.00000000E+00  0.00000000E+00  0.00000000E+00
  0.00000000E+00  0.00000000E+00  0.00000000E+00
  0.00000000E+00  0.00000000E+00  0.00000000E+00
  0.00000000E+00  0.00000000E+00  0.00000000E+00
  0.00000000E+00  0.00000000E+00  0.00000000E+00
  0.00000000E+00  0.00000000E+00  0.00000000E+00
  0.00000000E+00  0.00000000E+00  0.00000000E+00
  0.00000000E+00  0.00000000E+00  0.00000000E+00
  0.00000000E+00  0.00000000E+00  0.00000000E+00
  0.00000000E+00  0.00000000E+00  0.00000000E+00
  0.00000000E+00  0.00000000E+00  0.00000000E+00
  0.00000000E+00  0.00000000E+00  0.00000000E+00
  0.00000000E+00  0.00000000E+00  0.00000000E+00
  0.00000000E+00  0.00000000E+00  0.00000000E+00
  0.00000000E+00  0.00000000E+00  0.00000000E+00
  0.00000000E+00  0.00000000E+00  0.00000000E+00
```

Optimized structure of the most stable single Na-adsorbed-3x3 MoPC supercell

```
Na1 Mo9 P9 C9
   1.00000000000000
    10.2927129999999991   -0.0000040000000000    0.0000010000000000
    -5.1463599999999996    8.9137489999999993   -0.0000010000000000
     0.0000020000000000   -0.0000010000000000   20.4009040000000006
   C    Mo   P    Na
     9     9     9     1
Selective dynamics
Direct
  0.0000845020814308 -0.0000985736645543  0.4673948605831360
  0.0001100540283046  0.3335213701921953  0.4673797973843400
  0.0003735191993241  0.6668569735601326  0.4682758617381901
  0.3331403691936943 -0.0003765534760006  0.4682733853383036
  0.3352442008587128  0.3342761176117214  0.4668024885299528
  0.3331551518860935  0.6668439624263713  0.4683071086127339
  0.6664733926395967 -0.0001179706101363  0.4673821699434127
  0.6657104416018480  0.3342844900890551  0.4667629852956504
  0.6657155978242788  0.6647447948786686  0.4668092961687894
  0.2224539578362205  0.1115880061438455  0.4846977496524734
  0.2224548055094376  0.4441906420310906  0.4847063483200927
  0.2222276114753005  0.7777700821497185  0.4865635697135903
```

```
 0.5557933814922780  0.1115745812059626  0.4846684847149765
 0.5555487844436459  0.4444383723686815  0.4858586165556553
 0.5558058919720261  0.7775392765164307  0.4847115350772307
 0.8888842047523032  0.1111107958992577  0.4836064932382682
 0.8884192868501374  0.4442029104448982  0.4846682334341850
 0.8884020862135622  0.7775352115268813  0.4847027839716724
-0.0004346426662229  0.0004332203366926  0.5605684472520029
-0.0004587101083221  0.3324900102921335  0.5605776062759101
-0.0022568633655985  0.6655194380218972  0.5615986781300044
 0.3344757743581985  0.0022561876520453  0.5615968973060218
 0.3252543416908871  0.3292532245605354  0.5611132935223830
 0.3344514171090190  0.6655383894207234  0.5616298725760086
 0.6675076961114013  0.0004571042027149  0.5605796878046672
 0.6706989969000087  0.3292935850482054  0.5610819118217542
 0.6707321572581979  0.6747269820441741  0.5611184257053035
 0.5555085928542275  0.4444333691266635  0.6541194113332581

 0.00000000E+00  0.00000000E+00  0.00000000E+00
 0.00000000E+00  0.00000000E+00  0.00000000E+00
 0.00000000E+00  0.00000000E+00  0.00000000E+00
 0.00000000E+00  0.00000000E+00  0.00000000E+00
 0.00000000E+00  0.00000000E+00  0.00000000E+00
 0.00000000E+00  0.00000000E+00  0.00000000E+00
 0.00000000E+00  0.00000000E+00  0.00000000E+00
 0.00000000E+00  0.00000000E+00  0.00000000E+00
 0.00000000E+00  0.00000000E+00  0.00000000E+00
 0.00000000E+00  0.00000000E+00  0.00000000E+00
 0.00000000E+00  0.00000000E+00  0.00000000E+00
 0.00000000E+00  0.00000000E+00  0.00000000E+00
 0.00000000E+00  0.00000000E+00  0.00000000E+00
 0.00000000E+00  0.00000000E+00  0.00000000E+00
 0.00000000E+00  0.00000000E+00  0.00000000E+00
 0.00000000E+00  0.00000000E+00  0.00000000E+00
 0.00000000E+00  0.00000000E+00  0.00000000E+00
 0.00000000E+00  0.00000000E+00  0.00000000E+00
 0.00000000E+00  0.00000000E+00  0.00000000E+00
 0.00000000E+00  0.00000000E+00  0.00000000E+00
 0.00000000E+00  0.00000000E+00  0.00000000E+00
 0.00000000E+00  0.00000000E+00  0.00000000E+00
 0.00000000E+00  0.00000000E+00  0.00000000E+00
 0.00000000E+00  0.00000000E+00  0.00000000E+00
 0.00000000E+00  0.00000000E+00  0.00000000E+00
 0.00000000E+00  0.00000000E+00  0.00000000E+00
 0.00000000E+00  0.00000000E+00  0.00000000E+00
 0.00000000E+00  0.00000000E+00  0.00000000E+00
```

Optimized structure of the Na_6MoPC configuration

```
Na54 Mo9 P9 C9
   1.00000000000000
    10.2927000000000000   -0.0000040000000000    0.0000010000000000
    -5.1463599999999996    8.9137500000000003   -0.0000010000000000
     0.0000020000000000   -0.0000010000000000   32.9993180000000024
   C     Mo    Na    P
     9     9    54     9
```

```
Selective dynamics
Direct
 -0.0000639157938703 -0.0000099257783124  0.4668926031762101
  0.9999035685604366  0.3337115537354706  0.4671925901250525
 -0.0000697421171045  0.6663523454172293  0.4671832418457759
  0.3335061864694550 -0.0000767806278634  0.4671931326764358
  0.3334980718984769  0.3337296415667914  0.4671982352522064
  0.3332611831276607  0.6666918380709528  0.4671605283923078
  0.6662434081372155 -0.0000053626802408  0.4671794630799014
  0.6665388560666147  0.3333430229203699  0.4672758125896249
  0.6661856646052112  0.6663877154448630  0.4671984067794461
  0.2219060121805456  0.1110114854935352  0.4873902572243304
  0.2221247245382860  0.4445031902891380  0.4874581537464087
  0.2221294118608273  0.7777544608026636  0.4874558611115545
  0.5553986959604073  0.4444943552723805  0.4874904590148859
  0.5554023779445795  0.1110052115907871  0.4875048290768139
  0.8888990294144210  0.1110192134628297  0.4873932752132482
  0.8888721179358553  0.4445105072661706  0.4875051714555647
  0.8889053155508115  0.7780142140704317  0.4873852002712333
  0.5554519581904627  0.7778094284757663  0.4874626387002920
  0.5557349999999985  0.7778840000000002  0.7727130000000031
  0.5556020000000004  0.4443580000000011  0.5940680000000000
  0.2222360000000023  0.4444450000000018  0.5940930000000009
  0.2222200000000001  0.7777969999999996  0.5941180000000017
  0.2219660000000019  0.1109920000000031  0.5941299999999998
  0.4443407220268061  0.5556363805959441  0.4120900477746625
  0.4443537064871368  0.8887806582757450  0.4120874232080177
  0.8887690000000035  0.1112049999999982  0.5941919999999996
  0.2222774525272257  0.1111500014496331  0.3197343290359880
  0.8889399999999981  0.1111459999999980  0.7726800000000011
  0.5556679999999972  0.4444220000000030  0.7727229999999992
  0.5556630000000027  0.1112770000000012  0.7727199999999996
  0.7778863962462352  0.5556951168281236  0.2270762723385496
  0.8889429999999976  0.7778259999999975  0.7726749999999996
  0.2222080000000020  0.4443550000000016  0.7727190000000022
  0.2222030000000004  0.7778890000000018  0.7727140000000006
  0.2222620000000006  0.1111450000000005  0.7726789999999966
  0.4444434193132140  0.5556516159772626  0.2270186209790651
  0.4444482195266198  0.8887984838525956  0.2270218829521364
  0.4443923153441787  0.2222034982861211  0.2270697005045297
  0.2222610771499902  0.7778423637543926  0.3196877783797592
  0.7778540488829453  0.8889348673242402  0.2270370932671625
  0.7778844129515201  0.2222086081743909  0.2270875546731245
  0.1111594121473085  0.8889261143780605  0.2270352364333298
  0.8888159999999985  0.4444289999999995  0.7727160000000026
  0.8888170000000031  0.4444620000000015  0.5940569999999994
  0.4443491086284295  0.2222218302841139  0.4121328796087966
  0.5556389999999993  0.7778229999999979  0.5940950000000029
  0.5556470243084055  0.7778341944026070  0.3196900922415434
  0.5556455262568458  0.4444572551530395  0.3197356167322564
  0.1111679999999993  0.2222480000000004  0.6830929999999995
  0.1111689999999967  0.8889240000000029  0.6830980000000011
  0.8889449949963569  0.1111448140385091  0.3197439642350815
  0.7778380000000027  0.2222539999999995  0.6830610000000021
  0.5556351342360719  0.1112170610656185  0.3197389241338709
  0.7778429999999972  0.8889220000000009  0.6831000000000031
```

```
 0.4444420000000022  0.2222250000000017  0.6830300000000022
 0.4444689999999980  0.8888230000000021  0.6830259999999981
 0.4444650000000010  0.5556339999999977  0.6830179999999970
 0.1111787560533659  0.2224455078180008  0.4120484797828312
 0.1111812381636528  0.8887895305072174  0.4120460222920855
 0.1111614178744187  0.5556108898161931  0.4120878598218118
 0.7778333258248381  0.5555660671601234  0.6836421575402221
 0.8888924108956026  0.4444705562804401  0.3197385737691623
 0.1112890834008930  0.5556446964124820  0.2270177772474288
 0.8889463260069970  0.7778247278744814  0.3197365373291985
 0.7777375850061347  0.5556147099556985  0.4121324602355295
 0.1111558846044074  0.2222444150193062  0.2270359846118578
 0.8888850000000019  0.7777850000000015  0.5941669999999988
 0.7775276430325155  0.8887828254729769  0.4120519262899280
 0.1112579999999994  0.5556240000000017  0.6830229999999986
 0.2222669265741006  0.4444537270075025  0.3196886860175129
 0.7777447236751086  0.2222056022214408  0.4121280789568448
 0.5555179999999993  0.1112010000000012  0.5940659999999980
 0.9999136182022100  0.3329873157694995  0.5318583916450315
 0.6665811338793618  0.3333368917070800  0.5318370163829402
 0.6669510673065761  0.0000100697568510  0.5318652455050896
 0.3332899571630920  0.6667041056469383  0.5318512863344246
 0.3328716189632236  0.3329538862959308  0.5318504558112533
 0.3328335386711243 -0.0000095184546761  0.5318526016619726
-0.0000775313428710  0.6670808850558596  0.5318540200514293
 0.6669510660483412  0.6670031374192690  0.5318535287467336
-0.0001206395338383  0.0000011287487152  0.5319022861872184
```

```
 0.00000000E+00  0.00000000E+00  0.00000000E+00
 0.00000000E+00  0.00000000E+00  0.00000000E+00
 0.00000000E+00  0.00000000E+00  0.00000000E+00
 0.00000000E+00  0.00000000E+00  0.00000000E+00
 0.00000000E+00  0.00000000E+00  0.00000000E+00
 0.00000000E+00  0.00000000E+00  0.00000000E+00
 0.00000000E+00  0.00000000E+00  0.00000000E+00
 0.00000000E+00  0.00000000E+00  0.00000000E+00
 0.00000000E+00  0.00000000E+00  0.00000000E+00
 0.00000000E+00  0.00000000E+00  0.00000000E+00
 0.00000000E+00  0.00000000E+00  0.00000000E+00
 0.00000000E+00  0.00000000E+00  0.00000000E+00
 0.00000000E+00  0.00000000E+00  0.00000000E+00
 0.00000000E+00  0.00000000E+00  0.00000000E+00
 0.00000000E+00  0.00000000E+00  0.00000000E+00
 0.00000000E+00  0.00000000E+00  0.00000000E+00
 0.00000000E+00  0.00000000E+00  0.00000000E+00
 0.00000000E+00  0.00000000E+00  0.00000000E+00
 0.00000000E+00  0.00000000E+00  0.00000000E+00
 0.00000000E+00  0.00000000E+00  0.00000000E+00
 0.00000000E+00  0.00000000E+00  0.00000000E+00
 0.00000000E+00  0.00000000E+00  0.00000000E+00
 0.00000000E+00  0.00000000E+00  0.00000000E+00
 0.00000000E+00  0.00000000E+00  0.00000000E+00
 0.00000000E+00  0.00000000E+00  0.00000000E+00
 0.00000000E+00  0.00000000E+00  0.00000000E+00
 0.00000000E+00  0.00000000E+00  0.00000000E+00
 0.00000000E+00  0.00000000E+00  0.00000000E+00
```

0.00000000E+00	0.00000000E+00	0.00000000E+00
0.00000000E+00	0.00000000E+00	0.00000000E+00
0.00000000E+00	0.00000000E+00	0.00000000E+00
0.00000000E+00	0.00000000E+00	0.00000000E+00
0.00000000E+00	0.00000000E+00	0.00000000E+00
0.00000000E+00	0.00000000E+00	0.00000000E+00
0.00000000E+00	0.00000000E+00	0.00000000E+00
0.00000000E+00	0.00000000E+00	0.00000000E+00
0.00000000E+00	0.00000000E+00	0.00000000E+00
0.00000000E+00	0.00000000E+00	0.00000000E+00
0.00000000E+00	0.00000000E+00	0.00000000E+00
0.00000000E+00	0.00000000E+00	0.00000000E+00
0.00000000E+00	0.00000000E+00	0.00000000E+00
0.00000000E+00	0.00000000E+00	0.00000000E+00
0.00000000E+00	0.00000000E+00	0.00000000E+00
0.00000000E+00	0.00000000E+00	0.00000000E+00
0.00000000E+00	0.00000000E+00	0.00000000E+00
0.00000000E+00	0.00000000E+00	0.00000000E+00
0.00000000E+00	0.00000000E+00	0.00000000E+00
0.00000000E+00	0.00000000E+00	0.00000000E+00
0.00000000E+00	0.00000000E+00	0.00000000E+00
0.00000000E+00	0.00000000E+00	0.00000000E+00
0.00000000E+00	0.00000000E+00	0.00000000E+00
0.00000000E+00	0.00000000E+00	0.00000000E+00
0.00000000E+00	0.00000000E+00	0.00000000E+00
0.00000000E+00	0.00000000E+00	0.00000000E+00
0.00000000E+00	0.00000000E+00	0.00000000E+00
0.00000000E+00	0.00000000E+00	0.00000000E+00
0.00000000E+00	0.00000000E+00	0.00000000E+00
0.00000000E+00	0.00000000E+00	0.00000000E+00
0.00000000E+00	0.00000000E+00	0.00000000E+00
0.00000000E+00	0.00000000E+00	0.00000000E+00
0.00000000E+00	0.00000000E+00	0.00000000E+00
0.00000000E+00	0.00000000E+00	0.00000000E+00
0.00000000E+00	0.00000000E+00	0.00000000E+00
0.00000000E+00	0.00000000E+00	0.00000000E+00
0.00000000E+00	0.00000000E+00	0.00000000E+00
0.00000000E+00	0.00000000E+00	0.00000000E+00
0.00000000E+00	0.00000000E+00	0.00000000E+00
0.00000000E+00	0.00000000E+00	0.00000000E+00
0.00000000E+00	0.00000000E+00	0.00000000E+00
0.00000000E+00	0.00000000E+00	0.00000000E+00
0.00000000E+00	0.00000000E+00	0.00000000E+00
0.00000000E+00	0.00000000E+00	0.00000000E+00
0.00000000E+00	0.00000000E+00	0.00000000E+00
0.00000000E+00	0.00000000E+00	0.00000000E+00
0.00000000E+00	0.00000000E+00	0.00000000E+00
0.00000000E+00	0.00000000E+00	0.00000000E+00
0.00000000E+00	0.00000000E+00	0.00000000E+00
0.00000000E+00	0.00000000E+00	0.00000000E+00
0.00000000E+00	0.00000000E+00	0.00000000E+00
0.00000000E+00	0.00000000E+00	0.00000000E+00
0.00000000E+00	0.00000000E+00	0.00000000E+00
